# "Random" gentamicin concentrations do not predict trough levels in neonates receiving once daily fixed dose regimens

**DOI:** 10.1186/1471-2431-6-8

**Published:** 2006-03-17

**Authors:** Elaine M Boyle, Isobel Brookes, Kathy Nye, Mike Watkinson, F Andrew I Riordan

**Affiliations:** 1 Department of Child Health, Birmingham Heartlands Hospital, UK; 2 Health Protection Agency, West Midlands Public Health Laboratory, Birmingham Heartlands Hospital, UK

## Abstract

**Background:**

Monitoring plasma gentamicin concentrations in neonates 24 hours after a once daily dose (4 mg/kg) often necessitates additional blood sampling. In adults a nomogram has been developed enabling evaluation of gentamicin doses by sampling concentrations with other blood tests, 4 – 16 hours after administration. We attempted to develop a similar nomogram for neonates.

**Methods:**

In addition to standard 24 hour sampling to monitor trough concentrations, one additional "random" gentamicin concentration was measured in each of 50 neonates <4 days of age (median gestation 33 weeks [28–41]), when other blood samples were clinically necessary, 4 – 20 hours after gentamicin administration. 24 hour concentrations of >1 mg/L were considered high, and an indication to extend the dosing interval.

**Results:**

Highest correlation (r^2 ^= 0.51) of plasma gentamicin concentration against time (4 to 20 hours) was with logarithmic regression. A line drawn 0.5 mg/L below the true regression line resulted in all babies with 24 hr gentamicin concentrations >1 mg/L having the additional "random" test result above that line, i.e. 100% sensitivity for 24 hour concentrations>1 mg/L, though only 58% specificity. Having created the nomogram, 39 further babies (median gestation 34 weeks [28–41]), were studied and results tested against the nomogram. In this validation group, sensitivity of the nomogram for 24 hr concentrations >1 mg/L was 92%; specificity 14%, positive predictive value 66%, and negative predictive value 50%.

Prematurity (≤ 37 weeks) was a more sensitive (94%) and specific (61%) indicator of high 24-hour concentrations. 62 (87%) of 71 preterm babies had high 24-hour concentrations.

**Conclusion:**

It was not possible to construct a nomogram to predict gentamicin concentrations at 24 hours in neonates with a variety of gestational ages. Dosage tailored to gestation with monitoring of trough concentrations remains management of choice.

## Background

Aminoglycosides are widely considered to be the treatment of choice in newborn infants with suspected or proven sepsis. Gentamicin remains one of the most commonly used aminoglycosides [1]. It is usually given in combination with a β-lactam antibiotic to provide optimum cover against organisms most frequently responsible for infections during the neonatal period. However, a significant drawback in the use of aminoglycosides is their narrow therapeutic index, the potential for ototoxicity and nephrotoxicity and the need for monitoring of drug concentrations. In many cases, antibiotics are given to high-risk infants until infection is excluded, rather than to treat proven sepsis. Such courses may only last 48 hours or until the cultures are definitely negative and therefore early measurement of levels is preferable if a safe dosing regimen is to be achieved.

The search for the safest and most effective regimen for gentamicin administration in the newborn has led to exploration of varied doses and dosing intervals [2]. Suggested doses have ranged from 2 to 5 mg/kg/dose, given every 8–36 hours with target trough concentrations of 0.5–2 mg/L [3].

Traditionally, infants received multiple daily doses of gentamicin. There has been a move towards high dose, long interval treatment with once daily administration in adults [3-7]. This approach has been adopted in view of the improved efficacy achieved with higher peak concentrations, the prolonged duration of the post-antibiotic effect and similar or lower incidence of toxicity when compared with traditional dosing [3,6]. It has been shown to be a cost-effective method and more convenient than multiple administration [8].

Once daily aminoglycosides are also used in neonates [9]. However because of the long half-life of aminoglycosides in neonates this once daily regimen is more like steady state dosing achieved by 8 hourly dosing in adults. Our unit moved to once daily dosage some years ago.

This in turn presented the problem of finding the most appropriate way of monitoring to avoid nephrotoxicity and ototoxicity [10]. Third dose monitoring is commonly used, but this does not allow early modification of dose or time interval if high concentrations are detected, particularly in those babies given once daily aminoglycosides who might have only a short 48 hour course of treatment before negative cultures are confirmed. Monitoring before the third dose will thus be when the drug is being discontinued.

A nomogram has been developed for use in adults receiving once-daily gentamicin. This allows evaluation of gentamicin concentrations by sampling at any time between 6 and 14 hours after administration, to predict whether or not it is safe to give the next antibiotic dose [11]. If such a nomogram were available for use in the newborn it would have a number of advantages: avoiding potentially toxic concentrations of gentamicin with the second dose; avoiding delay in giving the second dose; reducing the number of needlesticks for infants by taking samples with other necessary blood tests; being able to process most samples during normal working hours; and avoiding the need for 24 hour trough samples.

### Aim of the study

The aim of this study was to develop and validate a simple and user-friendly nomogram to predict 24 hour trough concentrations reliably across a range of gestational ages, allowing modification of the dose at the earliest opportunity. We hypothesised that it would be possible to predict 24 hour trough concentrations from a single convenience sample taken between 4 and 20 hours after the first dose.

## Methods

The study included infants of birth weight ≥ 1000 g admitted to the Neonatal Unit of Birmingham Heartlands Hospital within the first four days of life, with increased risk for, suspected or proven sepsis. Infants received gentamicin 4 mg/kg once daily. Trough gentamicin concentrations were taken at 24 hours after the first dose, before a steady state was reached. For this reason, we aimed to achieve trough gentamicin concentrations of ≤ 1 mg/L. Clinical decisions about dosage alteration were based on this concentration. Infants <1000 g do not routinely receive aminoglycosides on our unit.

1 mg/l or less was considered as an acceptable trough gentamicin concentration. A second additional "random" sample for gentamicin concentration was taken from each baby between 4 and 20 hours after the first antibiotic dose, at a time when a blood sample was required for other clinical reasons. The additional sample was not truly "random" as its timing was dictated by the need for other blood tests, but nevertheless we have used the word "random" to signify this test and its results. Infants whose creatinine levels rose after birth or who had suffered birth asphyxia (requiring resuscitation for more than 10 minutes) were excluded from the study since they may have abnormal renal function.

Gentamicin concentrations were measured using a fluorescence polarization immunoassay (Abbott TDx, Abbott Laboratories, Queenborough, Kent, UK), with reagents supplied by Biostat Diagnostics Systems (Biostat Ltd, Stockport, Cheshire, UK).

Results from this derivation cohort of 50 babies were used to create a nomogram. A second cohort of babies was then recruited for validation of the nomogram.

Ethical approval for this research was obtained from the East Birmingham Research Ethics Committee. Verbal consent was obtained from parents of all the infants included in the study.

## Results

### Derivation cohort

Fifty babies with a median gestation of 33 weeks (range 28 – 41 weeks), and birth weight 2314 (1160 – 4640) grams, had a single "random" blood sample taken between 4 and 20 hours post gentamicin administration as well as a sample at 24 hours for trough gentamicin concentration.

**Figure 1:**
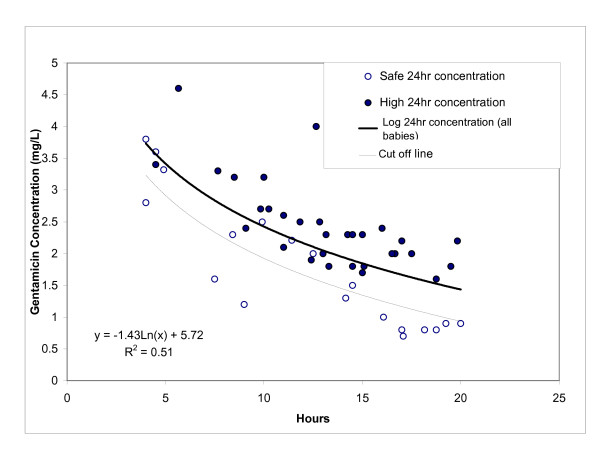
**Derivation Cohort: "Random" Gentamicin Concentration v Time**. The equation is given for the regression line which is the heavier of the two lines on the graph. The cut off line is the lighter and lower line (see Text). Safe 24 hr concentration = ≤ 1 mg/l. High 24 hr concentration = >1 mg/l.

Figure 1 shows the results. Thirty-one of the 50 babies had a 24 hr trough gentamicin concentration > 1 mg/L. The best regression of "random" gentamicin concentration against time after first dose for all 50 babies was logarithmic, with a regression coefficient (r) of 0.71 (95% CI 0.54, 0.83).

Nine of the 31 babies with 24 hour trough gentamicin levels >1 mg/l had a "random" concentration below the regression line. If this regression line were to be used as the cut off for predicting 24 hour concentrations, its sensitivity would be 84% (16/19), positive predictive value 64% (16/25) and specificity 71% (22/31). However, as the purpose of the nomogram would be to predict babies with gentamicin concentrations at 24 hours of <1 mg/l (ie. those babies who could be given a second dose without waiting for a 24 hour trough result), the positive predictive value of the cut off line would need to be 100%. To achieve this, the cut off line was drawn parallel to, but 0.5 mg/l below the regression line. All the babies with high 24-hour concentrations then had "random" values above this cut off line, i.e. its positive predictive value for babies with 24 hour concentrations <1 mg/l was 100%. However, 8 of the 19 babies with 24 hour gentamicin concentrations <1 mg/l were above this line, giving a sensitivity of only 58%.

### Validation cohort

**Figure 2:**
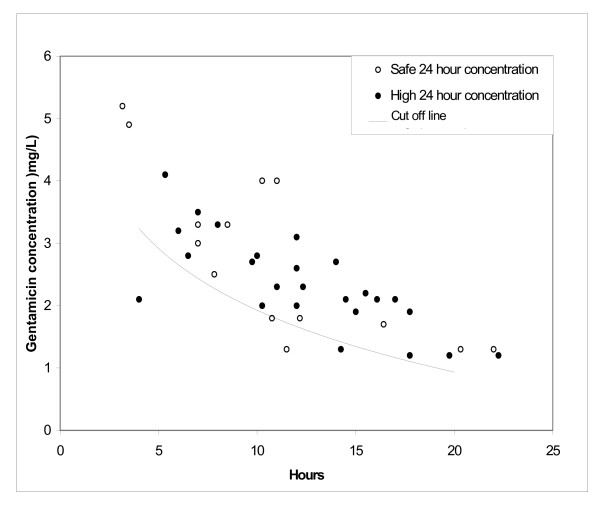
**Validation Cohort: "Random" Gentamicin Concentration v Time**. "Random" gentamicin concentrations from 39 validation babies plotted against the cut off line derived from Figure 1 (see Text). Safe 24 hr concentration = ≤ 1 mg/l. High 24 hr concentration = >1 mg/l.

To see if this cut off line could be used to predict 24 hour gentamicin concentrations <1 mg/l consistently, a further 39 babies had "random" and 24 hour gentamicin assays taken as described above. Their median (range) gestation was 34 (28–41) weeks, and birth weight 2380 (1120–4080) grams, not significantly different from the derivation cohort. Figure 2 shows the results against the cut off line. The sensitivity of the cut off line was 92% (23/25), specificity 14% (2/14), positive predictive value 66% (23/35) and negative predictive value 50% (2/4). Had the original regression line been used, the sensitivity would have been 50% and the positive predictive value 47%.

## Analysis by gestation

As the nomogram failed to be a useful predictor of trough gentamicin concentrations <1 mg/l, we re-analysed the data. Table 1 shows the 24-hour gentamicin concentration against gestation for all 89 babies. A trough gentamicin level <1 mg/l was significantly associated with increasing gestational age (P < 0.0001 by χ^2^). Table 2 shows the performance characteristics for predicting trough gentamicin concentrations <1 mg/l for the nomogram and for gestational age. Even in term babies (> 37 weeks), using gestation as a 'test' for 24 hour gentamicin levels <1 mg/l after a dose of 4 mg/Kg would only have a positive predictive value of 78% (14/18). With only 6% of babies less than 33 weeks having a 24 hour gentamicin concentration <1 mg/l, it is apparent that the dosing interval would need to be extended for the lowest gestations, and, in some cases, right up to 37 weeks.

**Table 1:** Proportion of trough gentamicin levels <1 mg/l in neonates given once daily gentamicin, by gestation.

Gestation (completed weeks)	n	24 hour gentamicin concentration <1 mg/l (%)
28 – 32	31	2 (6)
33 – 37	40	17 (42)
>37	18	14 (78)

**Table 2:** Performance characteristics of nomogram for predicting safe trough gentamicin levels (<1mg/l) in neonates given once daily gentamicin (4mg/kg)

	n	Sensitivity (%)	Specificity (%)	Positive Predictive Value (%)	Negative Predictive Value (%)
Derivation	50	100	58	79	100
Validation	39	92	14	66	50
Gestation >37 weeks	89	94	61	78	87

## Discussion

There is increasing support for the use of once daily aminoglycosides in adults and children [12]. Monitoring of once-daily aminoglycoside therapy in adults can now be done using a single serum level taken 6 to 14 hours after the aminoglycoside dose [11,13]. In adults with normal renal function this can be done using a nomogram. We were unable to make a similar, clinically useful nomogram to predict trough aminoglycoside concentrations for infants receiving once-daily gentamicin. In our study, gestational age was a better predictor of trough aminoglycoside concentration.

Many factors may influence trough aminoglycoside levels in neonates (gestational age, volume of distribution, clearance and half life). The most important seem to be gestational age and dose interval [10,14,15]. Chronological age with maturation of renal function and changes in body composition after birth may also be important [2,16]. We only studied infants in the first 4 days of life and excluded those below 1000 grams. However there was still a marked variation in gentamicin concentrations in these infants. We had expected that this variation in function would not affect the development of the nomogram, in that babies with good renal clearance of gentamicin would have had a "random" concentration below the cut off line and a 24 concentration < 1 mg/L. Equally those with less good renal function would have had a lower clearance of gentamicin and high "random" and 24 hour concentrations. Theoretically this should have dealt with variations in renal function due to gestation, postnatal age and disease. This proved not to be so.

Given the variation in aminoglycoside concentrations, it might be suggested that a larger number of subjects should have been studied to derive our nomogram. However the adult nomogram was devised using only 20 patients, 14 of whom had normal renal function [11]. The adult nomogram was then validated on a further 57 patients. Our study shows the importance of validating nomograms for once-daily aminoglycoside use for each group of patients.

A "safe" trough aminoglycoside concentration in those receiving once-daily dosing has not yet been clearly defined, especially in neonates [2]. There is little evidence to support the presently accepted "therapeutic range" in adults on multiple daily doses [17], and even less evidence in neonates [18]. In once-daily dosing in adults, some authors suggest trough concentrations of less than 2 mg/L are acceptable [11], whilst others suggest less than 1 mg/L [13]. We chose to use a concentration 1 mg/L or less as an acceptable trough in our study, because we were measuring concentrations after the first dose. The elimination of aminoglycosides in neonates is much slower than in adults, so accumulation of the drug will occur even with once-daily dosing. Results after a single dose will therefore be different from those obtained at a steady state. This low trough concentration therefore allows for accumulation of gentamicin with subsequent doses.

When should trough aminoglycoside levels be measured in neonates on once daily regimens? Measuring levels before the second dose will allow early identification of those at risk of possible toxicity and appropriate alteration in dose. If levels are not measured until before the third dose many neonates on 24 or 36 hourly dosing regimens will have stopped the antibiotic before this level is due. This result will thus be of little use.

Some suggest that it may not be necessary to measure levels in neonates given aminoglycosides once daily for 48 hours [2]. However given the variation in levels seen in our study it would seem prudent to measure levels on all neonates given aminoglycosides [19].

One implication of our study is that, for neonates below 32 weeks gestation receiving gentamicin 4 mg/kg, the dosing interval should be greater than 24 hours. Other authors have suggested this [7,15], but studies confirming the safety and efficacy of these doses in preterm infants are necessary. Clinical practice on our neonatal unit has changed, so that infants of less than 32 weeks gestation now receive gentamicin every 36 hours. The other implication is that all neonates need to have aminoglycoside concentrations measured because of the marked individual variation of results.

When suitable dosing regimens for once-daily aminoglycosides have been designed for preterm infants, it may be possible to construct nomograms to allow monitoring by "random" levels. This will require a larger number of infants to produce a number of nomograms for different gestations and postnatal ages or a nomogram with more than one cut-off line. Alternatively a multiple regression model using other factors might be needed. However we wanted to develop a simple nomogram for all infants.

Previous nomograms designed to aid gentamicin dosing in neonates have either failed to achieve gentamicin levels in the target range [20] or not been validated [21]. We were unable to construct a single, clinically useful nomogram to predict aminoglycoside concentrations for infants of varying gestations, given once daily gentamicin.

## Competing interests

The author(s) declare that they have no competing interests.

## Authors' contributions

EB participated in the design of the study, recruited infants to the study, did the statistical analysis and drafted the manuscript

IF recruited infants to the study, helped with the statistical analysis and edited the manuscript

KN participated in the design of the study, supervised the gentamicin assays and helped write the manuscript.

MW participated in the design of the study, recruited infants to the study, helped with the statistical analysis and edited the manuscript

FAIR participated in the design of the study and revised the manuscript

## Pre-publication history

The pre-publication history for this paper can be accessed here:



## References

[B1] McCracken GH, Nelson JD (1983). Antimicrobial Therapy for Newborns. Grune & Stratton.

[B2] Chattopadhyay B (2002). Newborns and gentamicin – how much and how often?. J Antimicrob Chemother.

[B3] Murphy JE, Austin ML, Frye RF (1998). Evaluation of gentamicin pharmacokinetics and dosing protocols in 195 neonates. Am J Health Syst Pharm.

[B4] Skopnik H, Wallraf R, Nies B, Troster K, Heimann G (1992). Pharmacokinetics and antibacterial activity of daily gentamicin. Arch Dis Child.

[B5] Prins JM, Buller HR, Kuijper EJ, Tange RA, Speelman P (1993). Once versus thrice daily gentamicin in patients with serious infections. Lancet.

[B6] Davies MW, Cartwright DW (1998). Gentamicin dosage intervals in neonates: longer dosage interval – less toxicity. J Paediatr Child Health.

[B7] Lundergan FS, Glasscock GF, Kim EH, Cohen RS (1999). Once-daily gentamicin dosing in newborn infants. Pediatrics.

[B8] Thureen PJ, Reiter PD, Gresores A, Stolpman NM, Kawato K, Hall DM (1999). Once- versus twice-daily gentamicin dosing in neonates >/=34 Weeks' gestation: cost-effectiveness analyses. Pediatrics.

[B9] de Alba RC, Gomez CE, Manzanares SC, Rodriguez LJ, Arreaza LL, Saenz VP (1998). Once daily gentamicin dosing in neonates. Pediatr Infect Dis J.

[B10] Mulhall A, de Louvois J, Hurley R (1983). Incidence of potentially toxic concentrations of gentamicin in the neonate. Arch Dis Child.

[B11] Nicolau DP, Freeman CD, Belliveau PP, Nightingale CH, Ross JW, Quintiliani R (1995). Experience with a Once-Daily Aminoglycoside Program Administered to 2,184 Adult Patients. Antimicrob Agents Chemother.

[B12] Barza M, Ioannidis JP, Cappelleri JC, Lau J (1996). Single or multiple daily doses of aminoglycosides: a meta-analysis. BMJ.

[B13] Begg EJ, Barclay ML, Duffull SB (1995). A suggested approach to once-daily aminoglycoside dosing. Br J Clin Pharmacol.

[B14] Keyes PS, Johnson CK, Rawlins TD (1989). Predictors of trough serum gentamicin concentrations in neonates. Am J Dis Child.

[B15] de Hoog M, Schoemaker RC, Mouton JW, van den Anker JN (1997). Tobramycin population pharmacokinetics in neonates. Clin Pharmacol Ther.

[B16] van den Anker JN (1996). Pharmacokinetics and renal function in preterm infants. Acta Paediatr.

[B17] McCormack JP, Jewesson PJ (1992). A critical reevaluation of the "therapeutic range" of aminoglycosides. Clin Infect Dis.

[B18] Rylance G (1983). Incidence of potentially toxic concentration of gentamicin in the neonate (Commentary). Arch Dis Child.

[B19] Nestaas E, Bangstad HJ, Sandvik L, Wathne KO (2005). Aminoglycoside extended interval dosing in neonates is safe and effective: a meta-analysis. Arch Dis Child Fetal Neonatal Ed.

[B20] Thomson AH, Campbell KC, Kelman AW (1990). Evaluation of six gentamicin nomograms using a bayesian parameter estimation program. Ther Drug Monit.

[B21] DiCenzo R, Forrest A, Slish JC, Cole C, Guillet R (2003). A gentamicin pharmacokinetic population model and once-daily dosing algorithm for neonates. Pharmacotherapy.

